# Draft genome sequence of *Ruoffia tabacinasalis* isolated from a bovine nasal swab: a novel member of the bovine nasal microbiota

**DOI:** 10.1128/mra.00087-24

**Published:** 2024-07-31

**Authors:** Samantha Howe, Xiaoyuan Wei, Jasna Kovac, Jiangchao Zhao

**Affiliations:** 1 Department of Animal Science, University of Arkansas, Fayetteville, Arkansas, USA; 2 Department of Food Science, Pennsylvania State University, State College, Pennsylvania, USA; Wellesley College Department of Biological Sciences, Wellesley, Massachusetts, USA

**Keywords:** whole-genome sequencing, nasal microbiota, bovine

## Abstract

We report the isolation and draft genome sequence of *Ruoffia tabacinasalis,* a novel member of the bovine nasal microbiota. The genome, which is estimated to be 90.5% complete, is composed of one contig comprising 2,363,349 bp with a GC content of 36.66%.

## ANNOUNCEMENT

Bovine respiratory disease (BRD) is the most devastating disease affecting American cattle producers, and research has illustrated a strong connection between the respiratory microbiota and BRD. Regardless, historically, most research regarding BRD and the respiratory microbiota has focused on the four major opportunistic pathogens (1). Commensal members of the bovine respiratory microbiota were isolated from nasal swabs. jzb001 was isolated from the nasal swab of a healthy steer from a research feedlot in West Texas (34.966086647966726,–101.80246082318041). A 1:10 dilution of swab buffer was plated onto *Mycoplasma* base agar (Criterion, Hardy Diagnostics, Santa Maria, CA, USA) + *Mycoplasma* supplement (Difco, BD, Sparks, MD, USA) inside a BSL-2 certified BioSafety Cabinet, and jzb001 was isolated after 72 hours of aerobic incubation at 37°C. *Ruoffia tabacinasalis* (formerly *Facklamia tabacinasalis*) was initially isolated and identified as a tobacco powder contaminant (2) and was recently reclassified as *R. tabacinasalis* (3). To our knowledge, this is the first time *R. tabacinasalis* has been isolated from the bovine nasal cavity.

For whole-genome sequencing, jzb001 was cultured overnight at 37°C on brain heart infusion agar. Three loops of overnight culture (originating from a single colony) were suspended in phosphate-buffered saline and vortexed in a bead beater at 3,500 rpm for 2 minutes for genomic DNA isolation using the Qiagen QIAamp DNA Blood Mini Kit (Qiagen, Hilden, Germany) following the manufacturer’s instructions. Next, the Rapid Barcoding Library Preparation Kit and AMPure XP beads were used to prepare the sequencing library (Oxford Nanopore Technologies). Sequencing was performed using the Oxford Nanopore MinION Mk1C sequencer with the flow cell R9.4.1 (FLO-MIN106D) for 30 hours. Guppy (version 6.1.2) was used for basecalling and trimming of DNA sequencing data obtained from the sequencer (4). The circular genome was assembled with Flye (version 2.9) (5) and polished with Racon (version 1.5.0) (6). Quality was assessed using QUAST (version 5.0.2) (7). The estimated *N*_50_ for jzb001 was 2.36 Mb (Table 1), and one contig was generated. Default parameters were used unless otherwise noted.

**TABLE 1:** jzb001 draft genome sequence metrics and results

Metric	Result	Tool	
Number of contigs	1	QUAST	
GC content	36.66%	QUAST	
Total size	2,363,349 bp	QUAST	
*N*_50_	2,363,349 bp	QUAST	
Coverage/depth	89×	Flye	
Estimated completeness	90.5%	Busco	
Predicted genes	2,307 CDS (total), 49 tRNA, 15 rRNA, 4 ncRNA, and 124 pseudogenes	PGAP	
Virulence factors	None detected	VFDB	
Antimicrobial resistance (AMR) genes	tet(M), ANT (6)-Ia	CARD	

**Fig 1:**
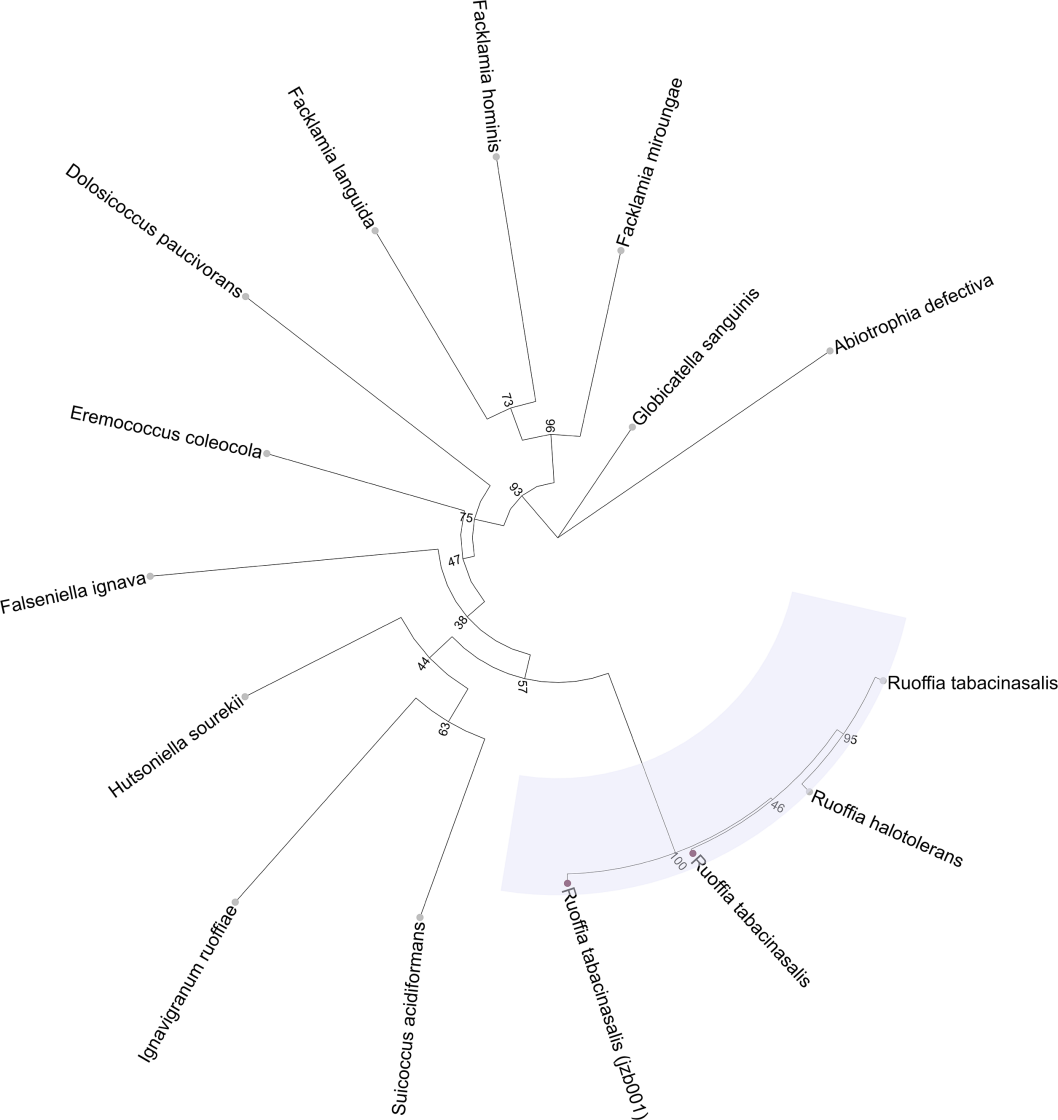
SNP-based unrooted phylogenetic tree of jzb001 and select members of the Aerococcaceae family. A consensus tree was constructed based on 100 bootstraps. Node labels indicate bootstrap values. Other sequences were acquired from NCBI RefSeq. Light purple indicates *Ruoffia* clade. Purple tip point indicates jzb001 and its closest match according to GTDBTk. Best fit model: GTR+F+I+G4; consensus tree log-likelihood: −15,106.811.

jzb001’s genome was annotated using the NCBI Prokaryotic Genome Annotation Pipeline (version 6.6) (8) (Table 1), and GTDBtk (version 2.1.0), along with its database (R207_v2), was used for taxonomic classification (9). jzb001 was identified as *R. tabacinasalis* [fastani_reference: GCF_005864045.1; average nucleotide identity (ANI): 96.5; alignment fraction: 0.82] using only ANI. As the above genome was not the *R. tabacinasalis* type strain, taxonomic assignment was confirmed by comparing jzb001 to the *R. tabacinasalis* type strain (GCF_015863285.1) using fastANI (version 1.33) (10) (ANI: 95.7). Busco (version 5.5) (11) (lineage: Lactobacillales_odb10) was used to estimate genome completeness (Table 1). PhaME (version 1.0.5) (12) was used to calculate the core genome SNPs of jzb001 and members of the Aerococcaceae family [reference: *Globicatella sanguinis* (GCF_002847845.2)]. The SNP alignment was used to reconstruct an unrooted phylogenetic tree using IQ-TREE (version 2.2.5) (13, 14), which was visualized using the ggtree R package (version 3.6.2) (15) (Fig. 1). Virulence factors were detected using the Virulence Factor Database (accessed 13 December 2023) (16, 17). AMR genes were detected using the Comprehensive Antibiotic Resistance Database (version 3.2.8) (18, 19) (Table 1).

## Data Availability

The assembled and annotated genome sequence was deposited at NCBI under the RefSeq accession number GCF_036866465.1, the BioProject accession number PRJNA1063262, and the BioSample accession number SAMN39331681. The raw reads were deposited into the NCBI SRA database under the run number SRR27966697. The other genome sequences utilized for SNP alignment and phylogenetic reconstruction include the following NCBI RefSeq accessions: GCF_013267415.1, GCF_002847845.2, GCF_023195815.2, GCF_003546865.1, GCF_002871685.1, GCF_000183205.1, GCF_000301035.1, GCF_000245795.1, GCF_012396555.1, GCF_002847645.1, GCF_000518205.1, GCF_014049385.1, GCF_005864045.1, and GCF_015863285.1.
